# Association between Use of Oral Anti-Diabetic Drugs and the Risk of Sepsis: A Nested Case-Control Study

**DOI:** 10.1038/srep15260

**Published:** 2015-10-14

**Authors:** Chia-Jen Shih, Yueh-Lin Wu, Pei-Wen Chao, Shu-Chen Kuo, Chih-Yu Yang, Szu-Yuan Li, Shuo-Ming Ou, Yung-Tai Chen

**Affiliations:** 1 School of Medicine, National Yang-Ming University, Taipei, Taiwan; 2 Department of Medicine, Taipei Veterans General Hospital, Yuanshan Branch, Yilan, Taiwan; 3 Division of Nephrology, Department of Medicine, Taipei City Hospital, Zhongxiao Branch Branch, Taipei, Taiwan; 4 Department of Anesthesiology, Wan Fang Hospital, Taipei Medical University, Taipei, Taiwan; 5 School of Medicine, Taipei Medical University, Taipei, Taiwan; 6 National Institute of Infectious Diseases and Vaccinology, National Health Research Institutes, Miaoli County, Taiwan; 7 Division of Infectious Diseases, Taipei Veterans General Hospital, Taipei, Taiwan; 8 Division of Nephrology, Department of Medicine, Taipei Veterans General Hospital, Taipei, Taiwan; 9 Institute of Clinical Medicine, National Yang-Ming University, Taipei, Taiwan; 10 Division of Nephrology, Department of Medicine, Taipei City Hospital, Heping Fuyou Branch, Taipei, Taiwan

## Abstract

Although oral antidiabetic drugs (OADs) have been associated with immunomodulation in preclinical studies, little is still known about the association between the use of OADs and the risk of sepsis. Using a cohort of patients, extracted from Taiwan’s National Health Insurance Research Database, with type 2 diabetes who were newly diagnosed between 2010 and 2012 and treated with OADs, we conducted a nested case-control study involving 43,015 cases (patients who were first hospitalized for sepsis) and 43,015 matched controls. Compared with non-use, metformin use was associated with a decreased risk of developing sepsis (adjusted odds ratio [OR] 0.80, 95% confidence interval [CI] 0.77–0.83, *P* < 0.001), but meglitinide (adjusted OR 1.32, 95% CI 1.25–1.40, *P* < 0.001) use was associated with the increased risk of developing sepsis. The risk for development of sepsis was also lower among current (adjusted OR 0.87, 95% CI 0.78–0.96) and recent (adjusted OR 0.83, 95% CI 0.73–0.94) thiazolidinedione users. Current or recent sulfonylurea use and dipeptidyl peptidase-4 inhibitor use were not significantly associated with the development of sepsis. Our results highlight the need to consider the potential pleiotropic effect of OADs against sepsis in addition to the lowering of blood glucose.

Between 1980 and 2008, the number of people with type 2 diabetes (T2D) worldwide increased from approximately 150 million to 350 million^1^. According to the World Health Organization, the global economic burden of T2D is tremendous, consuming 2.5–15% of countries’ primary healthcare budgets^2^. Till now, the use of oral antidiabetic drugs (OADs) remains the preferred pharmacological therapy due to many patients’ fear of insulin administration and its adverse effects, such as hypoglycemia and weight gain^3^,^4^.

Several classes of OAD are available on the market, including biguanide (metformin), sulfonylureas, alpha-glucosidase inhibitors, meglitinides, thiazolidinediones (TZDs), dipeptidyl peptidase-4 (DPP-4) inhibitors, and newer sodium-glucose cotransporter 2 (SGLT2) inhibitors. In addition to mediating glucose reduction, OADs have been associated with immunomodulation in preclinical studies^5^,^6^,^7^. In fact, patients with T2D are susceptible to infection and sepsis which may also impact on T2D lethality and medical costs in health systems; however, the possible pleiotropic effect of OADs on sepsis outcomes has not yet been well validated in large-scale clinical studies.

Previous studies^8^,^9^,^10^ exploring the association between OADs and sepsis have produced conflicting results, which may be attributed to methodological issues such as small samples, limited follow-up periods, unconfirmed diagnosis of infection events, unknown OAD exposure periods relative to sepsis onset, or the confounding effects of differences in diabetes severity between groups. To investigate whether susceptibility to sepsis differed among patients with T2D taking different classes of OAD, we used the Taiwan National Health Insurance Research Database (NHIRD) to conduct a nationwide nested case-control study that controlled for the effects of predisposing the host factors to sepsis.

## Methods

### Data sources

Medical care in Taiwan has been provided under Taiwan’s National Health Insurance (NHI) since 1995. This system covers more than 99% of Taiwan’s inhabitants for most medical expenses related to inpatient, outpatient, and emergency care, Chinese medicine, and dental services. For administrative and reimbursement purposes, the Bureau of the NHI audited patients’ diagnosis, procedure, and medication data to ensure correct coding and appropriateness; these data were recorded and stored in the NHIRD, which has been described in detail in our previous studies^11^,^12^. To examine OAD use among patients with T2D, we extracted the Longitudinal Cohort of Diabetes Patients dataset from the NHIRD. This dataset includes all available medical registry data for 120,000 patients with incident T2D per year during the period 1999–2012. This study was exempted from full review by the Institutional Review Board of Taipei City Hospital because it used de-identified and secondary claims data released by the NHIRD for research purposes.

### Study population

In this nationwide population-based study, we assembled a cohort of patients who received new diagnoses of diabetes between 2010 and 2012, as the marketing of DPP-4 inhibitors was approved in Taiwan in 2009. The definition of diabetes was based on the presence of one primary discharge diagnosis of diabetes (International Classification of Diseases, Ninth Revision, Clinical Modification [ICD-9-CM] code 250.x), two ambulatory visits with a diagnosis of diabetes (ICD-9-CM code 250.x), or use of any antidiabetic drug. The accuracy of coded DM diagnoses in the NHIRD has been validated^13^. The date of cohort entry was the first day on which a patient fulfilled the diabetes diagnostic criteria. At cohort entry, all individuals were required to be at least 20 years old and have baseline medical history for at least 5 years (i.e., 2005–2009) available in the NHIRD; these data were used for verification purposes. We excluded those who had been hospitalized for sepsis before cohort entry. Each subject was followed until the outcome of hospitalization for sepsis, death, loss to follow up, or the end of the study period (31 December 2012).

### Case definition and control selection

Because OAD exposure may be a time-dependent property associated with sepsis occurrence, we conducted a nested case-control analysis to estimate the odds ratios (ORs) for sepsis, comparing each OAD user with a nonuser of that drug. Cases were all patients hospitalized for sepsis (defined as a primary diagnosis of septicemia [ICD-9-CM code 038.x] plus the prescription of antibiotics) during the study period. We previously validated the accuracy of this definition of sepsis^11^. The index date was the day of the case’s hospital admission. For each case, we randomly selected a control matched according to age (±1 year), sex, month and year of cohort entry, level of urbanization, monthly income, Charlson Comorbidity Index score^14^, adapted Diabetes Complications Severity Index (aDCSI) score^15^, and duration of follow up.

### Exposure assessment

All OAD prescriptions for each subject in the year before the index date were identified. The OADs of primary interest in the present study were metformin, sulfonylureas, TZDs, meglitinides, and DPP-4 inhibitors. Given that preclinical studies^16^,^17^ have found that glibenclamide may have anti-inflammatory responses, but other sulfonylureas did not, we further stratified sulfonylureas into glibenclamide and non-glibenclamide sulfonylureas. For each OAD prescription, we collected the following information: dispensing date, drug type, quantity, and duration of drug supply. The pattern of OAD use was categorized as current (on index date), recent (≤30 days before index date), or past (31–365 days before index date).

### Statistical analysis

The baseline demographic characteristics of the cohort were analyzed using descriptive statistics. We conducted conditional logistic regression with adjustment for potential confounding factors, including OAD use, insulin use, and all other predefined comorbidities associated with the risk of sepsis (Table 1). ORs were computed to compare OAD exposure of cases and controls. The Microsoft SQL Server 2012 (Microsoft Corp., Redmond, Washington, USA) was used for data linkage, processing, and sampling. All analyses were performed using STATA statistical software (version 13.0; StataCorp., College Station, Texas, USA). Statistical significance was defined as *P* < 0.05.

## Results

A total of 43,015 cases and 43,015 matched controls were identified, with Table 1 showing their baseline characteristics. Hypertension, heart failure, cerebrovascular disease, and drug abuse were more prevalent among cases than among controls.

Table 2 presents the crude and adjusted ORs for the development of sepsis requiring hospitalization (cases) in association with OAD use compared with controls, after adjusting for all potential confounders in Table 1. Metformin use was associated with decreased odds of developing sepsis (adjusted OR 0.80, 95% confidence interval [CI] 0.77–0.83, *P* < 0.001), whereas sulfonylurea (adjusted OR 1.06, 95% CI 1.02–1.10, *P* = 0.001) and meglitinide (adjusted OR 1.32, 95% CI 1.25–1.40, *P* < 0.001) use were associated with increased odds of developing sepsis (Table 2). In addition, the timing of OAD use may be related with the onset of sepsis. Adjusted ORs for sepsis were 0.77 (95% CI 0.73–0.80) for current metformin use, 0.74 (95% CI 0.70–0.79) for recent metformin use, and 0.90 (95% CI 0.86–0.95) for past metformin use. Neither current sulfonylurea use (adjusted OR 1.03, 95% CI 0.98–1.08) nor recent (adjusted OR 0.97, 95% CI 0.91–1.03) sulfonylurea use significantly increased the risk of sepsis. The results remained similar when sulfonylureas were classified as either glibenclamide or non-glibenclamide (Supplementary Table 1). Current (adjusted OR 0.87, 95% CI 0.78–0.96) and recent (adjusted OR 0.83, 95% CI 0.73–0.94) TZD use significantly decreased the risk of sepsis.

The comparisons of metformin-based combined therapy versus metformin alone on the risk of sepsis are summarized in Table 3. A decreased risk of sepsis was consistently observed in patients taking metformin alone (adjusted OR 0.65, 95% CI 0.62–0.68) and metformin-based combination therapy with sulfonylureas (adjusted OR 0.72, 95% CI 0.69–0.75), TZDs (adjusted OR 0.51, 95% CI 0.41–0.64), DPP-4 inhibitors (adjusted OR 0.65, 95% CI 0.55–0.78), or meglitinides (adjusted OR 0.82, 95% CI 0.71–0.96).

## Discussion

To our knowledge, this is the first population-based, nested case-control study to examine the relationship between OAD use and the risk of hospitalization for sepsis.

We found that metformin use was associated with about 20% reduced risk of sepsis compared with nonuse. In contrast, meglitinides and sulfonylureas was associated with increased risk of future sepsis events, but this association was not evident among recent and current sulfonylurea users. The effects of DPP-4 inhibitors and TZDs on sepsis were neutral, but a reduced risk of sepsis occurrence was observed only in recent/current TZD users. Nevertheless, metformin-based OADs conferred a persistent benefit on the rate of hospitalization for sepsis.

In-vitro studies have found that metformin treatment had an inhibitory effect on mediators of sepsis, such as by limiting respiratory *Staphylococcus aureus* growth^8^ and tuberculosis^18^ and mucormycosis^19^ infection, and attenuating hepatitis B virus replication^20^. Similar to our findings, a Swedish population-based cohort study^10^ with a mean follow-up period of 3.9 years demonstrated that metformin treatment was associated with a reduced risk of composite outcomes of acidosis/serious infection (adjusted hazard ratio 0.85, 95% CI 0.74–0.97) in patients with T2D, independent of glycemic control, compared with those receiving other OADs (about 80% of which were sulfonylureas). Although relevant guidelines for diabetes treatment have suggested withdrawal from metformin for patients with sepsis due to concern about metformin-associated lactic acidosis^21^, this approach has been controversial because no proven evidence supports the increased risk of this condition among metformin users compared with users of other OADs^22^,^23^,^24^. In a single-center retrospective cohort study of 1,947 patients with septic emergent department events, a significant improvement in short-term survival of sepsis was noted for patients who had received metformin compared with those who had not (OR, 2.49; *P* < 0.01)^25^. Our nationwide study provided more evidence to support the association of metformin prescription with decreased risk of sepsis through the examination of patterns of past, recent, or current use. Furthermore, meglitinide prescriptions had the opposite effect on sepsis development, which appeared to be weaker for sulfonylurea users. Although investigation of the mechanism responsible for these relationships was beyond the scope of the present study, their propensities for insulin secretagogues by inhibiting the adenosine triphosphate–sensitive potassium channel in pancreatic β cells may also have off–target effects, which have been found to be related to impaired immune response against invading pathogens in preclinical studies^26^,^27^.

Recent/current, but not past, TZD prescription was associated with a modest reduction in sepsis risk, suggesting that this effect is immediate. Randomized controlled trials (RCTs)^28^,^29^ investigating the clinical effectiveness of add-on TZD therapy in patients with T2D showed no significant difference in additional infection risk between the TZD group and active controls; this is consistent with the findings of the present study. Only a modest potential benefit from TZD in sepsis onset may be offset in intention-to-treat analyses conducted in RCTs, as some patients were lost to follow up or withdrew from the medication during the follow-up period. In contrast, a meta-analysis^8^ of 13 trials showed that TZD use was associated with greater risks of upper and lower respiratory-tract infections, but low (<2% overall) event rates of sepsis and differences in follow-up periods (1–5 years) among trials may have affected the accuracy of estimates of incident sepsis events.

No association between DPP-4 inhibitors and sepsis risk was observed in the present study. DPP-4 inhibition may have pleiotropic effects, modulating the immune response by binding DPP-4 receptors of immune cells^30^ or culprit pathogens, such as coronavirus and hepatitis C virus^31^,^32^. The balance between immune inhibition and anti-inflammation may be responsible for infection risk in DPP-4 inhibitor users. In the context of weighing the pros and cons of DPP-4 inhibitor use given the effects of these drugs on immune function, our results show that they have an insignificant influence of sepsis risk. A nested control study based on the World Health Organization Vigibase^9^ showed that DPP-4 inhibitor use was associated with a greater risk of infection compared with metformin use. However, this result should be interpreted with caution, as the imprecise definition of infection based on spontaneous reporting introduces reporting bias.

The strengths of the present study include the analysis of large case and control groups respectively representing the nationwide diabetes populations that had previously received OADs either with or without sepsis from 2010–2012, which thus minimized referral bias. Additionally, we investigated whether the impact of OADs on the occurrence of sepsis was immediate or persistent over time by considering OAD exposure intervals. Still, this study has a few potential limitations. First, it was retrospective and observational in nature, and so causality could not be established. Second, the diagnosis of diabetes and sepsis based on ICD-9 CM codes may have introduced misclassification bias; however, this bias could be non-differential and robust agreement between diagnoses established by coding and clinical criteria has been demonstrated elsewhere^11^,^13^. Third, the claims database did not include individual baseline data on glycemic control, such as HbA1c levels. Nonetheless, if the impact of OADs on sepsis outcome were mainly the result of the glucose-lowering effect, the tendency of ORs for different OADs should tend toward coherence. Therefore, it is unlikely that this unmeasured confounder biased the results, and its effects were minimized by adjusting for the aDCSI score. Lastly, data on potential confounders such as obesity, smoking habit, and alcohol consumption were also unavailable in the database.

In conclusion, metformin and recent or current TZD use were inversely associated with sepsis occurrence, whereas meglitinide use was positively associated with sepsis occurrence. As patients with T2D are predisposed to infection, the direct impacts of OADs on future sepsis events should be considered.

## Additional Information

**How to cite this article**: Shih, C.-J. *et al*. Association between Use of Oral Anti-Diabetic Drugs and the Risk of Sepsis: A Nested Case-Control Study. *Sci. Rep*. **5**, 15260; doi: 10.1038/srep15260 (2015).

## Supplementary Material

Supplementary Information

## Figures and Tables

**Table 1:** Characteristics of the Cases and Controls.

	Cases (n = 43,015)	Control (n = 43,015)	*P* value
Age, mean (SD), year	78.3 (47.2)	78.4 (47.1)	0.852
Male sex	23,105 (53.7)	23,105 (53.7)	>0.99
Monthly income			>0.99
Dependent	17,258 (40.1)	17,258 (40.1)	
0–19,100 NT dollars	7,140 (16.6)	7,140 (16.6)	
19,100–42,000 NT dollars	174,952 (406.7)	17,495 (40.7)	
>42,000NT dollars	1,122 (2.6)	1,122 (2.6)	
Urbanization*			>0.99
Level 1	13,439 (31.2)	13,439 (31.2)	
Level 2	27,862 (64.8)	27,862 (64.8)	
Level 3	1,557 (3.6)	1,557 (3.6)	
Level 4	157 (0.4)	157 (0.4)	
Charlson comorbidity index score†			
1	2,315 (5.4)	2,176 (5.1)	0.758
2	2,945 (6.8)	2,955 (6.9)	0.893
3	4,497 (10.5)	4,590 (10.7)	0.302
≧4	33,258 (77.3)	33,294 (77.4)	0.769
Adapted Diabetes Complications Severity Index score (SD)‡	4.0 (2.8)	4.0 (2.7)	0.166
0	4,880 (11.3)	4,749 (11.0)	0.157
1	4,403 (10.2)	4,532 (10.5)	0.149
2	5,104 (11.9)	5,110 (11.9)	0.950
3	5,809 (13.5)	5,898 (13.7)	0.376
4	5,233 (12.2)	5,271 (12.3)	0.692
≧5	17,586 (40.9)	17,455 (40.6)	0.363
Duration of diagnosis of diabetes mellitus, months (SD)	78.3 (47.2)	78.4 (47.1)	0.852
Other comorbidity			
Hypertension	36,913 (85.8)	36,678 (85.3)	0.023
Coronary artery disease	23,739 (55.2)	26,298 (61.1)	<0.001
Myocardial infarction	3,669 (8.5)	3,733 (8.7)	0.437
Heart failure	12,840 (29.9)	11,973 (27.8)	<0.001
Dyslipidemia	27,217 (63.3)	31,468 (73.2)	<0.001
Cerebrovascular disease	21,821 (50.7)	20,095 (46.7)	<0.001
Chronic liver disease	19,031 (44.2)	21,056 (49.0)	<0.001
Chronic kidney disease	13,883 (32.3)	15,099 (35.1)	<0.001
Autoimmune disease	2,474 (5.8)	3,079 (7.2)	<0.001
Drug abuse	2,169 (5.0)	1,774 (4.1)	<0.001

^*^Urbanization levels in Taiwan are divided into four strata according to the Taiwan National Health Research Institute publications. Level 1 designates the most urbanized areas, and level 4 designates the least urbanized areas.

^†^Charlson Comorbidity Index (CCI) score is used to determine overall systemic health. With each increased level of CCI score, there are stepwise increases in the cumulative mortality.

^‡^Adapted Diabetes Complications Severity Index is a 13-point scale from 7 complication categories: retinopathy, nephropathy, neuropathy, cerebrovascular, cardiovascular, peripheral vascular disease, and metabolic, ranging from each complication. Each complication produced a numeric score ranging from 0 to 2 (0 = no abnormality, 1 = some abnormality, 2 = severe abnormality).

**Table 2:** Crude and Adjusted Rate Ratios for the Risk of Hospitalization for Sepsis With Oral Antidiabetic Drugs.

	No. (%)		odds Ratio (95%CI)			
	Cases (n = 43,015)	Control (n = 43,015)	Crude	*P*Value	Adjusted*	*P*Value
No Metformin use†	26,430 (61.4)	25,062 (58.3)	1		1	
Metformin use						
Any‡	16,585 (38.6)	17,953 (41.7)	0.87 (0.94–0.89)	<0.001	0.80 (0.77–0.83)	<0.001
Current§	6,755 (15.7)	7,828 (18.2)	0.81 (0.78–0.84)	<0.001	0.77 (0.73–0.80)	<0.001
Recent‖	3,647 (8.5)	4,532 (10.5)	0.76 (0.72–0.79)	<0.001	0.74 (0.70–0.79)	<0.001
Past¶	6,183 (14.4)	5,593 (13.0)	1.04 (1.00–1.08)	0.060	0.90 (0.86–0.95)	<0.001
No DPP-4 inhibitor use†	39,492 (91.8)	39,739 (92.4)	1		1	
DPP-4 use						
Any‡	3,523 (8.2)	3,276 (7.6)	1.09 (1.03–1.14)	0.001	1.01 (0.95–1.06)	0.842
Current§	1,148 (2.7)	1,152 (2.7)	1.01 (0.93–1.09)	<0.890	0.97 (0.89–1.07)	0.543
Recent‖	897 (2.1)	887 (2.1)	1.02 (0.93–1.12)	0.657	1.06 (0.95–1.18)	0.289
Past¶	1,478 (3.4)	1,237 (2.9)	1.21 (1.12–1.30)	<0.001	1.01 (0.92–1.10)	0.903
No Sulfonylurea use†	27,733 (64.5)	27,687 (64.4)	1		1	
Sulfonylurea use						
Any‡	15,282 (35.5)	15,328 (35.6)	1.00 (0.97–1.02)	0.736	1.06 (1.03–1.10)	0.001
Current§	6,364 (14.8)	6,964 (16.2)	0.91 (0.88–0.95)	<0.001	1.03 (0.98–1.08)	0.220
Recent‖	3,132 (7.3)	3,648 (8.5)	0.86 (0.81–0.90)	<0.001	0.97 (0.91–1.03)	0.288
Past¶	5,786 (13.5)	4,716 (11.0)	1.22 (1.17–1.28)	<0.001	1.18 (1.12–1.24)	<0.001
No Meglitinide use†	39,329 (91.4)	40,330 (93.8)	1		1	
Meglitinide use						
Any‡	3,686 (8.6)	2,685 (6.2)	1.42 (1.35–1.50)	<0.001	1.32 (1.25–1.40)	<0.001
Current§	1,357 (3.2)	1,016 (2.4)	1.38 (1.27–1.50)	<0.001	1.29 (1.17–1.41)	<0.001
Recent‖	721 (1.7)	565 (1.3)	1.32 (1.18–1.47)	<0.001	1.28 (1.13–1.44)	<0.001
Past¶	1,608 (3.7)	1,104 (2.6)	1.51 (1.40–1.63)	<0.001	1.32 (1.22–1.44)	<0.001
No Thiazolidinedione use†	40,025 (93.0)	39,901 (92.8)	1		1	
Thiazolidinedione use						
Any‡	2,990 (7.0)	3,114 (7.2)	0.96 (0.91–1.01)	0.093	0.95 (0.89–1.01)	0.085
Current§	745 (1.7)	913 (2.1)	0.81 (0.74–0.90)	<0.001	0.87 (0.78–0.96)	0.009
Recent‖	534 (1.2)	731 (1.7)	0.73 (0.65–0.81)	<0.001	0.83 (0.73–0.94)	0.003
Past¶	1,711 (4.0)	1,470 (3.4)	1.16 (1.08–1.24)	<0.001	1.07 (0.98–1.16)	0.114

^*^Adjusted for oral antidiabetic drugs, insulin use, and all confounders in Table 1. Each level of Charlson Comorbidity Index and adapted Diabetes Complications Severity Index was calculated as a separate indicator variable.

^†^During the year prior to the index date.

^‡^Use of 1 prescription at any time prior to the index date.

^§^A prescription termination date (date of dispensation plus the day supply) overlapping with the index date.

^‖^A prescription termination date of 1 to 30 days before the index date.

^¶^A prescription termination date of 31 to 365 days before the index date.

**Table 3:** Crude and Adjusted Rate Ratios for the Risk of Hospitalization for Sepsis with Metformin-Based Combination Therapy.

	No. (%)		Rate Ratio (95% CI)			
Current exposure	Cases (n = 43,015)	Control (n = 43,015)	Crude	*P* Value	Adjusted*	*P* Value
Metformin Alone	3,135 (7.3)	4,467 (10.4)	0.64 (0.61–0.67)	<0.001	0.65 (0.62–0.68)	<0.001
Metformin+ Sulfonylurea	4,929 (11.5)	6,265 (14.6)	0.72 (0.69–0.75)	<0.001	0.72 (0.69–0.75)	<0.001
Metformin+ Thiazolidinedione	132 (0.3)	241 (0.6)	0.50 (0.41–0.62)	<0.001	0.51 (0.41–0.64)	<0.001
Metformin+ DPP-4 inhibitors	215 (0.5)	300 (0.7)	0.65 (0.55–0.78)	<0.001	0.65 (0.55–0.78)	<0.001
Metformin+ Meglitinides	341 (0.8)	380 (0.9)	0.82 (0.71–0.96)	0.010	0.82 (0.71–0.96)	0.012

^*^Adjusted for all confounders in Table 1. Each level of Charlson Comorbidity Index and adapted Diabetes Complications Severity Index was calculated as a separate indicator variable.
