# Supplementary material for: Association between Use of Oral Anti-Diabetic Drugs and the Risk of Sepsis: A Nested Case-Control Study
Source: Sci Rep. 2015 Oct 14;5:15260. doi: 10.1038/srep15260 (PMC4604480; doi:10.1038/srep15260)
Supplement: Supplementary Information [file srep15260-s1.doc]

**Association between Use of Oral Anti-Diabetic Drugs and the Risk of Sepsis: A Nested Case-Control Study**

Chia-Jen Shih, MD; Yueh-Lin Wu, MD; Pei-Wen Chao, MD; Shu-Chen Kuo, MD; Chih-Yu Yang, MD, PhD; Szu-Yuan Li, MD, PhD; Shuo-Ming Ou, MD; Yung-Tai Chen, MD

| **Supplementary Table 1. Crude and Adjusted Rate Ratios for the Risk of Hospitalization for Sepsis With different sulfonylureas (SU)** | | | | | | | | |
| --- | --- | --- | --- | --- | --- | --- | --- | --- |
|  | **No. (%)** | |  | **Odds Ratio (95% CI)** | | | | |
|  | **Cases**  **(n=43,015)** | **Control**  **(n=43,015)** |  | **Crude** | ***P***  **Value** |  | **Adjusted^*^** | ***P***  **Value** |
| **No Glibenclamide use** ^†^ | 40,366 (93.8) | 40,702 (94.6) |  | 1 [Reference] | |  | 1 [Reference] | |
| **Glibenclamide use** |  |  |  |  |  |  |  |  |
| **Any** ^‡^ | 2,649 (6.2) | 2,313 (5.4) |  | 1.16 (1.09-1.23) | <0.001 |  | 1.07 (1.03-1.11) | <0.001 |
| **Current** ^§^ | 797 (1.9) | 779 (1.8) |  | 1.04 (0.94-1.14) | 0.495 |  | 1.17 (1.05-1.30) | 0.006 |
| **Recent** ^‖^ | 400 (0.9) | 430 (1.0) |  | 0.94 (0.82-1.08) | 0.391 |  | 0.98 (0.84-1.14) | 0.785 |
| **Past** ^¶^ | 1,452 (3.4) | 1,104 (2.6) |  | 1.33 (1.22-1.44) | <0.001 |  | 1.26 (1.16-1.37) | <0.001 |
| **No other-SU use ^†^** | 29,261 (68.0) | 29,225 (67.9) |  | 1 [Reference] | |  | 1 [Reference] | |
| **Other SU use** |  |  |  |  |  |  |  |  |
| **Any** ^‡^ | 13,754 (32.0) | 13,790 (32.1) |  | 1.00 (0.97-1.03) | 0.787 |  | 1.32 (1.25-1.40) | <0.001 |
| **Current** ^§^ | 5,608 (13.0) | 6,209 (14.4) |  | 0.90 (0.87-0.94) | <0.001 |  | 1.29 (1.17-1.41) | <0.001 |
| **Recent** ^‖^ | 2,817 (6.5) | 3,266 (7.6) |  | 0.86 (0.82-0.91) | <0.001 |  | 1.28 (1.13-1.45) | <0.001 |
| **Past** ^¶^ | 5,329 (12.4) | 4,315 (10.0) |  | 1.23 (1.18-1.29) | <0.001 |  | 1.33 (1.22-1.45) | <0.001 |
| **^*^** Adjusted for oral antidiabetic drugs, insulin use, and all confounders in Table 1.  ^†^ During the year prior to the index date.  ^‡^ Use of 1 prescription at any time prior to the index date.  ^§^ A prescription termination date (date of dispensation plus the day supply) overlapping with the index date.  ^‖^ A prescription termination date of 1 to 30 days before the index date.  ^¶^ A prescription termination date of 31 to 365 days before the index date. | | | | | | | | |
